# Giant Right Pulmonary Artery Aneurysm and Patent Ductus Arteriosus in an Adult Patient With Eisenmenger's Syndrome

**DOI:** 10.1016/j.opresp.2022.100210

**Published:** 2022-09-16

**Authors:** Andres Obeso

**Affiliations:** Department of Thoracic Surgery, Hospital Clínico Universitario de Santiago de Compostela, Santiago de Compostela, Spain

A 47-year-old symptomatic male patient with history of idiopathic pulmonary arterial hypertension (PAH) since 2004 was referred to our institution for evaluation. The patient complained of hemoptysis and chest pain since five months ago. On-admission pulmonary artery pressure was 51 mmHg. Axial (Fig. 1A) and coronal (Fig. 1B) contrast-enhanced chest computed tomography with 3D reconstruction (Fig. 1D) showed a 5.5 cm × 8 cm aneurysm of the right pulmonary artery. Additionally oblique plane (Fig. 1C) revealed a non-previous detected patent ductus arteriosus (PDA). This congenital heart disease turned out to be responsible for the Eisenmenger's syndrome. No other cardiac abnormalities were detected by echocardiography. After a meticulous counseling and information, the patient finally rejected any surgical treatment and he was lost.

**Fig. 1:**
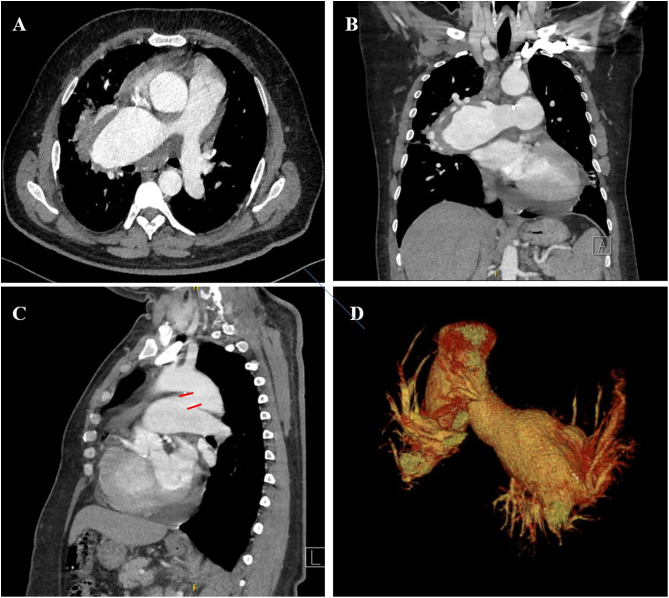
Contrast-enhanced chest CT scan shows an aneurysm of the right pulmonary artery in axial (A) and coronal (B) plane. Oblique plane (C) shows a patent ductus arteriosus marked with red lines. Image D represents a 3D reconstruction of the aneurysmal lesion.

Pulmonary artery aneurysm associated to PDA and Eisenmenger's syndrome is a very unfrequent clinical entity and only a few cases have been reported in the literature.^1^ Due to this very low incidence, no clear management consensus have been established. In patients with significant PAH or connective tissue disease, which increases the risk of rupture, surgical treatment must be always considered, specially in patients with symptoms or progressive changes.^2^

## Authors’ contributions

The author has made substantial contribution to all of the following: (1) provide care to the patient as part of the surgical team (2) write the entire manuscript and edition of the images (3) review the content and final approval of the version. (4) Submit the manuscript to the journal.

## Informed consent

Informed consent was obtained from the patient for publication of the clinical data and images present in this manuscript.

## Funding

The author declares that he did not receive any fees or funding for the development of the clinical image presented.

## Conflicts of interest

The author declares that he has no known competing financial interests or personal relationships that could have appeared to influence the work reported in this paper.
